# A Fuzzy Analytic Hierarchy Process and Cooperative Game Theory Combined Multiple Mobile Robot Navigation Algorithm

**DOI:** 10.3390/s20102827

**Published:** 2020-05-16

**Authors:** Changwon Kim, Jong-Seob Won

**Affiliations:** 1 Daegu Research Center for Medical Devices and Rehabilitation, Korea Institute of Machinery and Materials, Daegu 42994, Korea; cwkim@kimm.re.kr; 2 Department of Mechanical and Automotive Engineering, Jeonju University, Jeonju 55069, Korea

**Keywords:** fuzzy-based AHP (FAHP), multi-objective decision making, path planning, mobile robot

## Abstract

This study presents a multi-robot navigation strategy based on a multi-objective decision-making algorithm, the Fuzzy Analytic Hierarchy Process (FAHP). FAHP analytically selects an optimal position as a sub-goal among points on the sensing boundary of a mobile robot considering the following three objectives: the travel distance to the target, collision safety with obstacles, and the rotation of the robot to face the target. Alternative solutions are evaluated by quantifying the relative importance of the objectives. As the FAHP algorithm is insufficient for multi-robot navigation, cooperative game theory is added to improve it. The performance of the proposed multi-robot navigation algorithm is tested with up to 12 mobile robots in several simulation conditions, altering factors such as the number of operating robots and the warehouse layout.

## 1. Introduction

Many research works have been undertaken and have focused on multi-robot systems due to their advantages such as complex task accomplishment, faster task completion based on parallelism, and their redundancy-based increase of robustness [1]. Multiple mobile platforms could be potentially used in future; for example, in highly automated factories for logistics, unmanned parking facilities for autonomous parking, and even advanced hospitals. The application of mobile robots in various fields aims to improve safety as well as work efficiency. However, to maximize the efficiency of mobile robot-based services, the systems must have collision-free navigation capability to ensure safety. Additionally, traveling distance is an important factor to consider in terms of work efficiency. In a standardized or unchangeable environment, the production efficiency can be increased by taking the shortest distance and with the high-speed driving of the mobile robot. Finally, depending on the type of work objects, such as semiconductors or hazardous materials, it is necessary to control them sensitively to avoid sudden movements of the robot. In other words, it is necessary to consider various objectives simultaneously in mobile robot path planning. To this end, many path planning research works have focused on multi-objective optimization problems, taking into account various aspects instead of focusing only on driving distance or collision safety with obstacles. Castillo et al. [2] suggested a genetic algorithm to optimize the travel distance and travel difficulty of the path simultaneously. Masehian and Sedighizadeh [3] combined particle swarm optimization and a probability road map, considering shortness and smoothness as the optimization objectives. The non-dominated Sorting Genetic Algorithm II was modified by Ahmed and Deb [4], taking into account travel distance, safety, and path smoothness simultaneously. The follow-up studies [5,6,7,8,9] suggested multi-objective optimization-based path planning strategies. Kim and Langari [10] utilized the Analytic Hierarchy Process (AHP) to plan an optimal path of a mobile robot considering the distance to the target, collision safety, and rotation to the target under the preference of travel. AHP is a multi-purpose decision-making technique which was developed by Saaty [11] and has been applied to various decision-making fields such as offshore manufacturing plant location [12], the selection of a nuclear reactor type [13], mobile robot control [14], and Web service selection [15], and so on [16,17,18]. Unlike the aforementioned multi-objective optimization methods, which have determined weights to consider objectives, AHP allows the setting of relative importance for each objective based on the user’s preference. AHP can model a given problem simply and flexibly; using AHP, it is also possible to measure consistency in decision making and to check that appropriate decision making is possible. Also, AHP’s simple calculation process can contribute to improving computational efficiency in the creation of a mobile robot’s path [19]. Recently, improving on the weaknesses of AHP, Kim et al. [20] proposed a fuzzy-based AHP navigation algorithm for a single robot.

Most multi-robot research has focused mainly on two areas: task allocation and robot collision prevention. This study proposes a collision-free path plan for mobile robots and an algorithm for preventing collisions between robots. To plan a collision-free path, many studies on multiple robots have been conducted over several decades. By addressing uncertainties, Hennes et al. [21] suggested a collision avoidance algorithm for multi-robots using the velocity obstacle paradigm. However, because the velocity obstacle concept-based algorithm could lead to oscillations for a robot, Rashid et al. [22] introduced the reciprocal orientation algorithm. Palm et al. [23] combined the artificial potential field and fuzzy logic algorithm for multiple robot navigation. A hybrid algorithm that deals with both collision avoidance and the task allocation algorithm was proposed, using a genetic algorithm and $A^{*}$ algorithm, by Jose and Pratihar [24]. Claes and Tuyls [25] introduced a human-aware navigation algorithm based on the velocity obstacle to apply multiple robots in a situation in which human coworkers exist. Recently, reinforcement learning has been utilized in multi-robot areas. Fan et al. [26], Ma et al. [27], and Bae et al. [28] proposed a deep reinforcement learning-based multi-robot navigation strategy. Furthermore, game theory is utilized in multi-robot navigation and task allocation in [29,30,31,32].

This research develops a multi-objective optimization-based multi-robot collision-free navigation algorithm. FAHP-based optimal path planning is possible in a scenario in which one robot moves to a target position. However, in an environment in which multiple robots are co-working, it is essential to generate optimal paths without collision. Notably, cooperative game theory-based decision making is introduced to cope with the multi-robot navigation problem. To implement this, FAHP is combined with cooperative game theory (CTG). With the proposed algorithm, the mobile robot selects and moves to the next location via FAHP. When it encounters another robot within the sensing area, cooperative game theory determines the optimal movement location that does not collide with other robots. The performance of the proposed algorithm is demonstrated under numerical simulations under two different categories: in one, the number of working agents in an open space scenario is different; the other case is a fully automated warehouse scenario. In the warehouse scenarios, three different scenarios are assumed, which are likely to occur in a multi-robot operating environment. The robustness and collision avoidance function are observed, taking into account the uncertainties from the robot’s localization error and the sensor’s measurement error.

During the robot operation, FAHP keeps calculating to generate a path, and CGT selectively conducts calculations when multi-robots are encountered. Because one of the advantages of AHP is the simplicity of calculation and CGT is also able to make decisions with a low number of computations, the combined algorithms also have computational efficiency. Therefore, the main contribution of this research is the proposal of a novel multi-robot collision-free navigation algorithm by combining FAHP and CGT that requires a lower computing load. The remainder of the paper is organized as follows: Section 2 provides a detailed explanation of fuzzy-based AHP. The application of the FAHP-game theory hybrid algorithm to multiple mobile robot navigation is described in Section 3. In Section 4, the performance of the suggested navigation algorithm is demonstrated by sets of simulations. The paper is concluded with a discussion in Section 5.

## 2. Problem Description

In highly automated assembly lines, fully robotized smart farms, and hospital halls to serve patients, multiple mobile robots are employed for logistics and surveillance purposes. By applying multiple robot systems, it is possible to maximize work efficiency. However, since multiple robots move on each path in the same space, not only can collisions occur with each other, but also movement may be restricted by other robots. Additionally, in real-world situations, there is an inherent error in robot localization and sensor measurement. Due to these factors, work efficiency could be reduced. For this reason, this study aims to propose a method to prevent collisions between robots and increase driving distance efficiency in the operation of a multi-robot system.

In order to approach the multi-robot problem practically, our simulation designed scenarios that can occur in the warehouse layout. The main operating system allocates a task to each robot, and *N* mobile robots ($R_{1}$, $R_{2}$, ⋯, $R_{n}$, n≥2) transport materials from their initial position ($p_{i}$, *i* = 1, 2, …, n) to the target position ($t_{i}$, *i* = 1, 2, …, n). Once the initial position and the target position are given, the path of the robot is generated. In previous research [20], an optimal path planning method—FAHP—was developed. However, a collision between robots could occur using this method because the initial path did not consider the possibility of encountering another robot on the way to its destination. Since it is difficult to solve the multi-robot navigation problem with FAHP alone, this study aims to achieve the desired purpose by combining it with cooperative game theory. Therefore, the originality and novelty of this research come from the combination of FAHP and CGT for a multi-robot navigation problem.

In every movement to the target, each robot senses the environment via light detection and ranging (LiDAR) to find a possible moving position (candidates). Each candidate position is evaluated with respect to the objectives such as traveling distance, safety, and robot rotation. Finally, FAHP helps to decide an optimal position among candidates for movement by selecting the candidate that has the highest evaluating value. However, when multiple robots are working in the same space, it is quite difficult to predict the path of other robots. Furthermore, as the number of robots increases, it is almost impossible to predict the path of other robots. To cover this, a robot communication-based CTG concept is employed. Each robot has several strategies (moving candidates), and each strategy has its own payoff (evaluation points). By sharing (via communication) this information with other robots and keeping a safe distance, a Pareto optimal solution-based collision-free path is generated.

## 3. Fuzzy-Based Analytic Hierarchy Process

Mobile robot navigation is an essential task for successful mission accomplishment. If the environment of the navigation task is simple enough—i.e., moving a straight line—the solution is also straightforward. However, in a complex working environment with obstacles, workers, and other robots, many objects should be considered during navigation. Therefore, mobile robot navigation needs to take into account travel distance, collision safety, and even the smoothness of the travel. In other words, many objectives need to be considered simultaneously in mobile robot path planning. To this end, for decades, several types of research have been undertaken on multi-objective decision making (MODM) based mobile robot navigation [2,3,4,5,6,7,8,9,10]. In particular, Kim and Langari [10] utilized the Analytic Hierarchy Process (AHP), a famous MODM tool, to navigate a mobile robot to a target without collision. Saaty [11] introduced AHP. Moreover, a specific aspect of AHP-based navigation is that it can apply the user’s preference for decision making. AHP-based route planning follows the following procedures:

- Step 1: Model the problem as a hierarchy: the decision goal, the alternatives as solution candidates, and the objectives to evaluate the candidates.
- Step 2: Establish priorities among the considered objectives: define the relative importance of the objectives by comparing them in pairs using a nine-point scale.
- Step 3: Synthesize the user’s priorities to yield a set of overall priorities for the hierarchy.
- Step 4: Check the consistency of the decision making.
- Step 5: Evaluate the candidates considering the weighted importance matrix.

However, the conventional AHP has some drawbacks [33]: (1) the AHP method creates and deals with a very unbalanced scale of judgment; (2) the AHP method does not take into account the uncertainty associated with the mapping of one’s judgment to a number; (3) the ranking of the AHP method is somewhat imprecise; and (4) the subjective judgment, selection, and preference of decision-makers have a significant influence on the AHP results. As a result, a pair-wise comparison scale based on AHP is insufficient to explain uncertain conditions.

To overcome these weaknesses, fuzzy-based AHP (FAHP) has been suggested; in the mobile robot field, Kim et al. [20] utilized FAHP. As a modified version of AHP, Chang [34] proposed the extent analysis method for fuzzy AHP based on the fuzzy number. As defined in [35], a fuzzy number *M* on *R* is a triangular fuzzy number if its membership function $\mu_{M}\left(x\right)=:R\to \left[0,1\right]$ is equal to

(1) $$\mu_{M}\left(x\right)=\left\{\begin{array}{cc} \frac{x}{m-l}-\frac{l}{m-l}, & x\in [l,m]\\ \frac{x}{m-u}-\frac{u}{m-u}, & x\in [m,u]\\ 0, & \mathrm{otherwise} \end{array}\right.,$$

where l≤m≤u. Moreover, *l*, *m*, and *u* represent the lowest, middle, and the highest value of *M*, respectively. Table 1 shows the nine Fuzzified Satty’s scales for the triangular fuzzy number [36].

Implementing FAHP requires the following steps [34].

- Step 1: Definition of the relative importance among objectives.
  (2) $$\mathrm{RM}=\left[\begin{array}{ccc} O_{1}/O_{1} & O_{1}/O_{2} & O_{1}/O_{3}\\ O_{2}/O_{1} & O_{2}/O_{2} & O_{2}/O_{3}\\ O_{3}/O_{1} & O_{3}/O_{2} & O_{3}/O_{3} \end{array}\right]=\left[\begin{array}{ccc} 1 & a & b\\ 1/a & 1 & c\\ 1/b & 1/c & 1 \end{array}\right],$$
  where $O_{n}$ represents the *n*-th objectives. *RM* is defined based on the notion that the first objective is *a* times as important as the second objective while *b* times as important as the third objective. Furthermore, the second objective is *c* times as important as the third. The general form of the *RM* is
  (3) $$\mathrm{RM}=\left[\begin{array}{cccc} O_{1}/O_{1} & O_{1}/O_{2} & \cdots & O_{1}/O_{n}\\ O_{2}/O_{1} & O_{2}/O_{2} & \cdots & O_{2}/O_{n}\\ \vdots & \vdots & \ddots & \vdots \\ O_{m}/O_{1} & O_{m}/O_{2} & \cdots & O_{m}/O_{n} \end{array}\right].$$
- Step 2: Consistency check of the relative important matrix.

However, the *RM* could be inappropriate due to the limitation of Saaty’s [34] discrete nine-value scale and the inconsistency of human judgements. Therefore, the consistency of *RM* should be examined by an AHP-based consistency method. Saaty [11] proposed a method to measure inconsistency; Saaty proved that the largest eigenvalue of the *RM* is equal to the size of the matrix, i.e., $\lambda_{max}$ = *n*, under perfect consistency. It is also possible to estimate the departure from consistency by the consistency index (*CI*). Therefore, the *CI* is

(4) $$\mathrm{CI}=\frac{\lambda_{max}-n}{n-1},$$

*CI* is divided by the random consistency (*RC*) index given in Table 2 to obtain the consistency ratio (*CR*) as follows:

(5) $$\mathrm{CR}=\frac{\mathrm{CI}}{\mathrm{RC}}.$$

Saaty [37] states that the appropriate measure as denoted by the *CR* should not exceed 0.1. Only a case which meets this condition can be accepted; otherwise, another relative importance matrix, *FRM*, is assessed until the *CR* appropriately satisfies the condition *CR* < 0.1.

- Step 3: Fuzzification of the relative importance matrix.

Using the triangular fuzzy number, the fuzzified relative importance matrix of Equation (2) is defined as follows:

(6) $$\mathrm{FRM}=\left[\begin{array}{ccccccccc} {}[1 & 1 & 1] & [a-d & a & a+d] & [b-d & b & b+d]\\ {}[\frac{1}{a+d} & \frac{1}{a} & \frac{1}{a-d}] & [1 & 1 & 1] & [c-d & c & c+d]\\ {}[\frac{1}{b+d} & \frac{1}{b} & \frac{1}{b-d}] & [\frac{1}{c+d} & \frac{1}{c} & \frac{1}{c-d}] & [1 & 1 & 1] \end{array}\right],$$

where FRM is the fuzzified relative importance matrix.

- Step 4: Calculation of fuzzy synthetic extent.

The fuzzy synthetic extent [38] of FRM is calculated as follows:

(7) $$S_{i}=\sum_{j=1}^{m}FRM_{g_{i}}^{j}⊙{\left[\sum_{i=1}^{n}\sum_{j=1}^{m}FRM_{g_{i}}^{j}\right]}^{-1},$$

where $S_{i}$ is the *i*-th synthetic extent and all the $FRM_{g_{i}}^{j}$ values are triangular fuzzy numbers. The definition of the operator ⊙ is

(8) $$\left(l_{1},m_{1},u_{1}\right)⊙\left(l_{2},m_{2},u_{2}\right)=\left(l_{1}\times l_{2},m_{1}\times m_{2},u_{1}\times u_{2}\right),$$

(9) $${\left(l_{1},m_{1},u_{1}\right)}^{-1}=\left(\frac{1}{u_{1}},\frac{1}{m_{1}},\frac{1}{l_{1}}\right).$$

- Step 5: Calculation of weight vectors of FRM.

**Figure 1:**
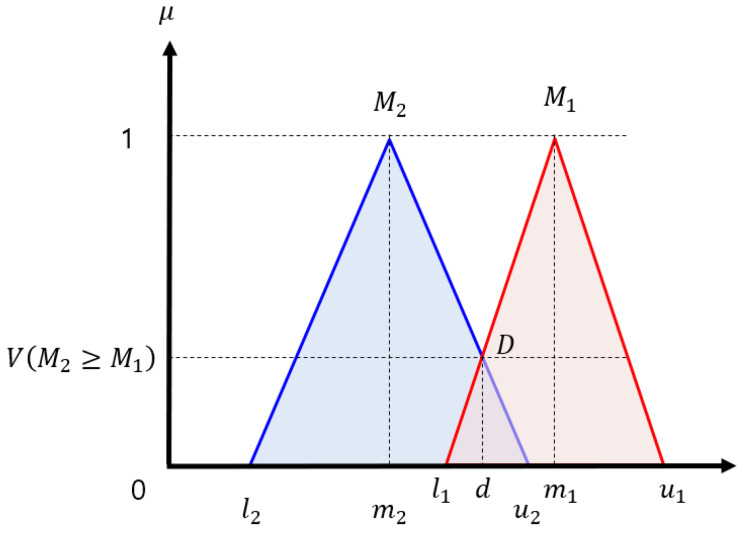
The intersection between $M_{1}$ and $M_{2}$ [39].

After the fuzzy synthetic extent is obtained, the weight vector of the defined objectives is derived. By the comparison principle of fuzzy numbers [33], the degree of possibility of $M_{2}=\left(l_{2},m_{2},u_{2}\right),\geq M_{1}=\left(l_{1},m_{1},u_{1}\right)$ is defined as

(10) $$V\left(M_{2}\geq M_{1}\right)=\underset{y\geq x}{\sup}\left[\min\;\left(\mu_{M_{1}}\left(x\right),\mu_{M_{2}}\left(y\right)\right)\right],$$

and Equation (10) is equivalently expressed as

(11) $$\begin{array}{cc} V(M_{2}\geq M_{1}) & =hgt\left(M_{1}\cap M_{2}\right)=\mu_{M_{1}}\left(d\right)\\ \; & =\left\{\begin{array}{cc} 1, & \;\mathrm{if}\;m_{2}\geq m_{1}\\ 0, & \;\mathrm{if}\;l_{1}\geq u_{2}\\ \frac{l_{1}-u_{2}}{\left(m_{2}-u_{2}\right)-\left(m_{1}-l_{1}\right)}, & \mathrm{otherwise} \end{array}\right. \end{array},$$

where *hgt* and *d* represent the highest intersection point and x coordinate of the two fuzzy numbers, respectively, as shown in Figure 1.

The degree of possibility for a convex fuzzy number to be greater than *k* convex fuzzy numbers $M_{i}\left(i=1,2,\cdots ,k\right)$ can be

(12) $$\begin{array}{c} V(M\geq M_{1},M_{2},M_{3},\ldots ,M_{k})\\ =\min\;V(M\geq M_{i}) \end{array}.$$

Assume that

(13) $$d^{{}^{\prime }}\left(O_{i}\right)=\min\;V\left(S_{i}\geq S_{k}\right),$$

for k=1,2,⋯,n;k≠i. Then, the weight vector is given by

(14) $$W^{{}^{\prime }}={\left(d^{{}^{\prime }}\left(O_{1}\right),d^{{}^{\prime }}\left(O_{2}\right),\ldots ,d^{{}^{\prime }}\left(O_{n}\right)\right)}^{T},$$

where $O_{i}\left(i=1,2,\cdots ,n\right)$ are *n* elements. By normalizing Equation (14), the normalized weight vector is given as follows:

(15) $$W_{obj}={\left(d\left(O_{1}\right),d\left(O_{2}\right),\ldots ,d\left(O_{n}\right)\right)}^{T}.$$

After the investigation of the consistency of the FRM, the given candidates are evaluated with respect to each objective. The objective based candidates evaluation matrix is given as follows:

(16) $$E{\left(O_{i}\right)}_{lm}=\frac{O_{i}\left(C_{l}\right)}{O_{i}\left(C_{m}\right)},$$

where $O_{i}\left(C_{l}\right)$ represents the score of *l*th candidate when the *i*th objective is considered, and l=1,2,⋯,γ, where γ denotes the number of candidates. Equation (16) is normalized, and the weighted candidate matrices of each corresponding objective are given by the following equations:

(17) $$E{\left(O_{i}\right)}_{norm(l)}=\left[\begin{array}{cccc} \frac{E{\left(O_{i}\right)}_{l1}}{\sum_{i=1}^{\gamma }E{\left(O_{i}\right)}_{l1}} & \frac{E{\left(O_{i}\right)}_{l2}}{\sum_{i=1}^{\gamma }E{\left(O_{i}\right)}_{l2}} & \cdots & \frac{E{\left(O_{i}\right)}_{l\gamma }}{\sum_{i=1}^{\gamma }E{\left(O_{i}\right)}_{l\gamma }} \end{array}\right].$$

The weighted candidate matrix of each objective is obtained by using the following equation:

(18) $$W_{candi}\left(O_{i}\right)=\left[\begin{array}{cccc} \frac{\sum_{m=1}^{\gamma }E{\left(O_{i}\right)}_{norm(1m)}}{\gamma } & \frac{\sum_{m=1}^{\gamma }E{\left(O_{i}\right)}_{norm(2m)}}{\gamma } & \cdots & \frac{\sum_{m=1}^{\gamma }E{\left(O_{i}\right)}_{norm(\gamma m)}}{\gamma } \end{array}\right].$$

All functions are considered to obtain the following weighted candidate matrix:

(19) $$W_{candi}=\left[\begin{array}{cccc} W_{candi}{\left(O_{1}\right)}^{T} & W_{candi}{\left(O_{2}\right)}^{T} & \cdots & W_{candi}{\left(O_{n}\right)}^{T} \end{array}\right].$$

A candidate that achieves the highest value is selected as the optimal solution by multiplying the two resulting matrices—using Equations (13) and (19)—and composed of weights as follows:

(20) $$Function^{*}=\underset{l}{\mathrm{argmax}}\left(W_{candi}\times W_{obj}^{T}\right).$$

## 4. Application of FAHP to Multi-Robot Collision-Free Navigation

**Figure 2:**
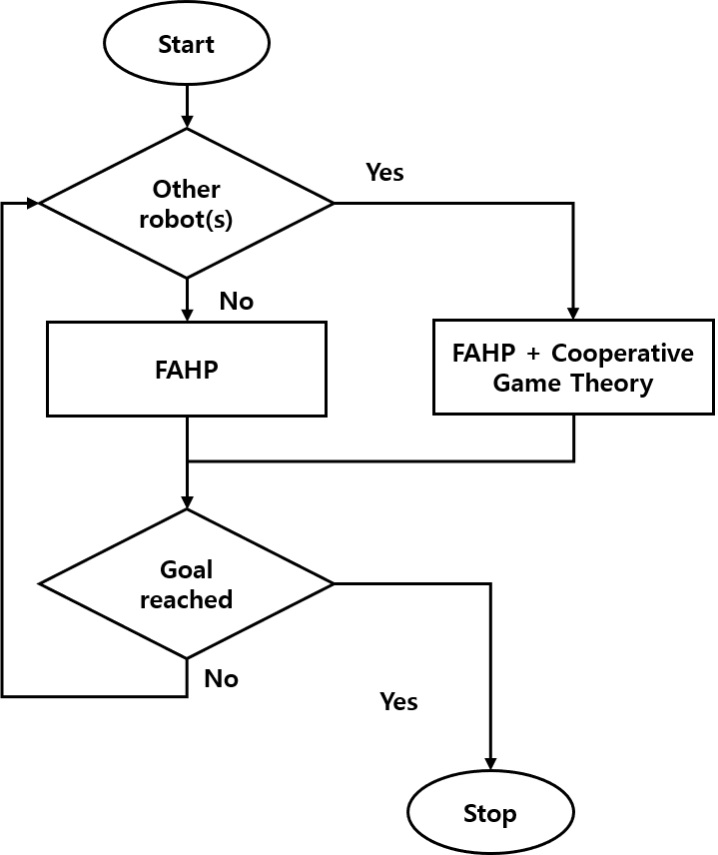
Multi-robot navigation strategy. FAHP: Fuzzy Analytic Hierarchy Process.

In this section, a multi-mobile robot navigation strategy is presented. In real robot operating environments, several mobile robots work together, interacting with each other. Sometimes, the robot encounters other robots in working boundaries, but sometimes they do not. Therefore, a multi-robot navigation strategy is necessary when two or more robots interact. Thus, the suggested navigation algorithm consists of the FAHP algorithm and FAHP–cooperative game theory (CGT) combined algorithm, as shown in Figure 2.

If no robots exist within the sensing boundary, the robot moves directly to the target with the FAHP navigation method. However, when the robot encounters other robots, it is assumed that all the robots share their information about the benefits obtained by adopting a satisfactory solution. From the communication among robots under the CGT framework, the optimal solution that can assign each robot’s action is selected.

### 4.1. FAHP Algorithm-Based Mobile Robot Navigation

**Figure 3:**
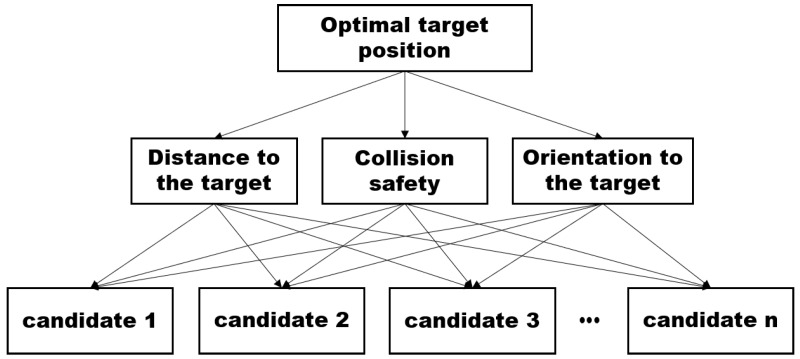
FAHP-based decision making structure.

In order to implement FAHP, the structure of decision making should be defined, as shown in Figure 3. The structure includes a final goal of decision making (selection of an optimal goal) as the highest level, considering factors to select a candidate as the middle level, and the candidate positions to which the robot will move as the lowest level [20]. In this research, the final goal of the FAHP is the selection of a sub-goal that is collision-free and in the direction of the target. Furthermore, the candidates sense boundary points. To select a solution among candidates, three objectives are considered: the distance to the target, collision safety, and orientation to the target. This is because the traveling distance helps to increase working efficiency, the safety score ensures collision-free operation, and the number of steep turns is involved in the damage to the transporting material. All the candidates are evaluated by the objectives, as explained in the previous section.

**Figure 4:**
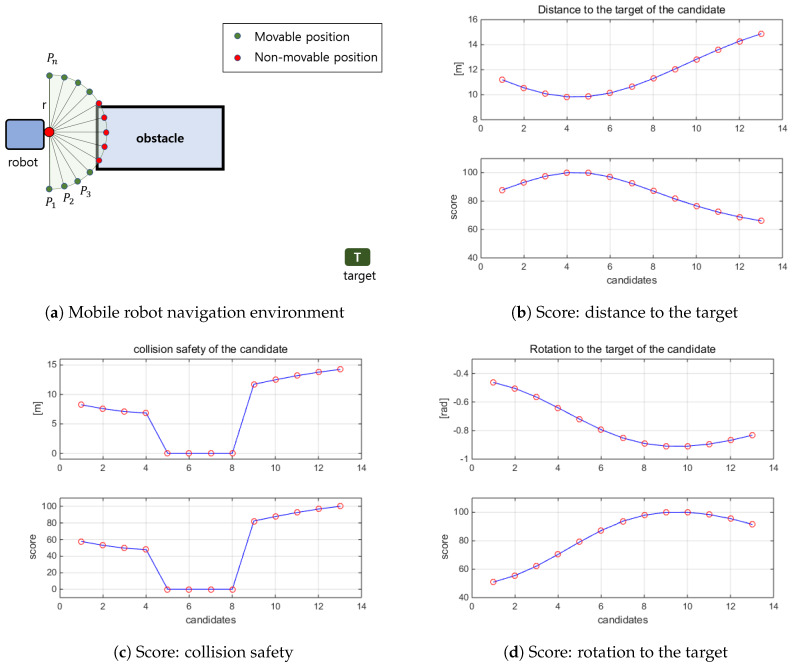
Candidate evaluation based on objectives.

Figure 4 displays an example of FAHP implementation for mobile robot navigation. The following are assumed to explain FAHP application: a mobile robot equipped with a LiDAR navigates to the target, as shown in Figure 4. The LiDAR has a resolution of pi/12 with detection range “r” in the angle range of −pi/2 to pi/2 from the robot’s local coordination system. Moreover, the endpoint of the sensing range is the next point to move to for the robot. The green dots represent where the robot can move, while the red dots are non-movable positions due to obstacles.

Implementing FAHP requires the organization of the decision-making hierarchy. The final goal is to select a point among candidates, $P_{1}$ and $P_{n}$. The three objectives are considered—distance to the target, collision safety, and rotation to the target—and the objectives are named $O_{1}$, $O_{2}$, and $O_{3}$, respectively. When all the candidates are evaluated by objectives, the scores of them are different, as shown in Figure 4b–d. In terms of distance to the target, $P_{4}$ is the shortest point among candidates, while $P_{n}$ is the safest solution because this point is the furthest from the obstacle. Moreover, after the robot moves to $P_{9}$, it rotates through the smallest angle to face the target. However, in multi-objective decision making, all the objectives are considered simultaneously under predefined preferences. In every movement cycle, the LiDAR detects the navigation environment and generates candidate positions for movement. Then, an optimal sub-target position is selected by means of FAHP.

### 4.2. FAHP and Cooperative Game Theory Combined Algorithm-Based Mobile Robot Navigation

A mobile robot can encounter other robots on its way to the target. If the paths of the interacting robots intersect with each other, collisions between robots cannot be avoided. In order to operate all the robots efficiently, FAHP is not sufficient because the LiDAR cannot predict the path of the other robots exactly. To this end, FAHP is combined with CGT. Game theory is a famous decision-making tool and has many branches. In this research, cooperative game theory is implemented to handle the multi-robot navigation problem, assuming all the robots communicate with each other. In the framework of game theory, the terminologies are defined in Table 3.

In order to implement game theory for the multi-robot navigation problem, *n* mobile robots are considered as the players in the game. Using FAHP, each robot makes a decision ($s_{i}$) regarding where to move, and this decision is considered as the strategy. Because multiple robots interact with each other, the decision of each robot affects the others. In other words, the payoff of the *i*-th robot ($u_{i}\left(s_{i},s_{i-1}\right)$) varies with the strategy set, **s**. The CGT guarantees a cooperative solution compared with other decision-making tools [29]. Therefore, the framework of a cooperative game is utilized, assuming that all the robots share their decisions and corresponding benefits (payoffs) with others. Furthermore, all the participants find a consensus to avoid collision and to find an optimal action to achieve each agent’s goal.

Since the main purpose of CGT is to find the final outcome, the domain of all possible outcomes, **U**, needs to be analyzed. In the CGT, no alternative strategy can improve all the players’ payoffs simultaneously. This property is called Pareto optimality [40] and is defined as follows [29]: “A set of payoffs from corresponding strategies $u^{*}=\left(u_{1},u_{2},\cdots ,u_{n}\right)$ is called ‘Pareto optimal’ if the inequalities $u_{i}\leq u_{i}^{*}$, i=1,2,⋯,n where at least one strict inequality does not have a solution for u.”

It is noted that the Pareto optimal solution shows that no player can increase their reward without reducing the reward of another player. The set of Pareto optimal solutions is called the Pareto boundary. Moreover, the Pareto optimal solution is not a unique solution but a set of these solutions [41]. However, the purpose of cooperative game theory is to decide a solution within the Pareto boundary. In order to solve the optimization problem, two approaches are commonly used: the first method is implemented by providing an offer and counter-offer among optimal boundaries until the players reach agreeable consensus, while in the second method, the Nash arbitration scheme is utilized to find an acceptable solution for all players. Particularly in multi-player games, where the number of participants is larger than two, the generalized axiom for the Nash arbitration method is applied as follows.

(21) $$u^{*}=\underset{u\in U}{\mathrm{argmin}}\left(\prod_{j}v_{j}\right),$$

where $v_{j}$ is defined as

(22) $$v^{j}=\left|d^{i}-u_{i}\right|,\;\left(u\in U\;with\;u\leq d\right),$$

where $d_{i}$ represents the best payoff for player *i* under a non-cooperative game. Equation (21) shows that the decision is made to minimize each player’s playoff loss. In the non-cooperative game, players select the best solution only for each player among $S_{i},1\leq i\leq n$, where each robot has its own strategy space. Assuming the *i*-th robot has *m* candidates and *j*-th robot has *l* candidates, the strategy spaces of each robot are given by $\left\{s_{i1},s_{i2},\ldots ,s_{im}\right\}$ and $\left\{s_{j1},s_{j2},\ldots ,s_{jl}\right\}$, respectively. In the mobile robot navigation problem, each strategy is matched to a point; i.e., $s_{im}$$\to P_{im}$. In order to apply game theory to mobile robot navigation, two aspects are considered; the payoff, which is defined as the FAHP calculation score (multiplication of Equations (13) and (19)), and the collision avoidance criteria, which arise from the investigation of collisions between robots. The second term is defined as follows:

(23) $$C_{ic_{i}jc_{j}}=\bar{P_{ic_{i}}P_{jc_{j}}},\;\left\{1\leq c_{i}\leq m,\;1\leq c_{j}\leq l,\;\mathrm{and}\;c_{i},c_{j}\in N\right\}$$

where $\bar{P_{ic_{i}}P_{jc_{j}}}$ represents the distance between two solution points, $P_{ic_{i}}$ and $P_{jc_{j}}$; i.e., the distance between the *i*-th robot’s $c_{i}$-th strategy and *j*-th robot’s $c_{j}$-th strategy. If the value of Equation (23) is less than the collision avoidance margin, $d_{min}$, a collision between the *i*-th and *j*-th robot occurs, and such conditions are excluded while seeking the solution using Equation (24). The payoff function $u_{i}$ is given as

(24) $$\begin{array}{c} u_{i}=\left\{\begin{array}{cc} W_{candi}\times W_{obj}^{T}, & \mathrm{if}\;C_{ic_{i}jc_{j}}\geq d_{min}\\ \mathrm{do}\;\mathrm{not}\;\mathrm{exist}, & \mathrm{otherwise} \end{array}\right. \end{array},$$

where $W_{candi}$ and $W_{obj}$ are Equations (13) and (19), respectively.

## 5. Simulation and Results

In this section, the performance of the suggested navigation algorithm is investigated under various conditions such as that of a single robot, multiple robots without obstacles, and warehouse layout-based multi-robot operation. Simulations are performed in the MATLAB environment. In the simulation, a LiDAR-equipped small AGV model with a radius of 1.1 m is assumed to cover an area of a commercial AGV; i.e., KUKA KMP 1500, 2000 ×800 (length × width). Furthermore, to show the robustness of the proposed algorithm for the robot’s localization uncertainty originating from a robot’s internal or external issues and sensor noise in application fields, a 3% random error following normal distribution was included in the robot’s position and sensing data.

### 5.1. Simulation I (FAHP-Based Single Mobile Robot Navigation)

Firstly, FAHP-based navigation is demonstrated with a single robot operating situation. This simulation was performed under a scenario in which the robot moved to the target position while avoiding the various obstacles in their path. In order to apply FAHP, two different relative importance (user’s preference) matrices are defined between the objectives (distance to the target, collision safety, and rotation to the target); i.e., one with the highest weight on the distance to the target, ${\mathrm{RM}}_{\mathrm{dist}}$, and the other on safety, ${\mathrm{RM}}_{\mathrm{safety}}$ as follows:

$${\mathrm{RM}}_{\mathrm{dist}}=\left[\begin{array}{ccc} 1 & 2 & 4\\ 1/2 & 1 & 1\\ 1/4 & 1 & 1 \end{array}\right]\;\mathrm{and}\;\;{\mathrm{RM}}_{\mathrm{safety}}=\left[\begin{array}{ccc} 1 & 1/4 & 2\\ 4 & 1 & 3\\ 1/2 & 1/3 & 1 \end{array}\right],$$

where ${\mathrm{RM}}_{\mathrm{dist}}$ shows that $O_{1}$ is twice as important as $O_{2}$ and four times as important as $O_{3}$, while $O_{2}$ is equally important as $O_{3}$. Furthermore, ${\mathrm{RM}}_{\mathrm{safety}}$ means that the collision safety ($O_{2}$) and rotation ($O_{3}$) are four times and half as important as the distance to the target ($O_{1}$), respectively. Collision safety is three times as important as rotation to the target.

**Figure 5:**
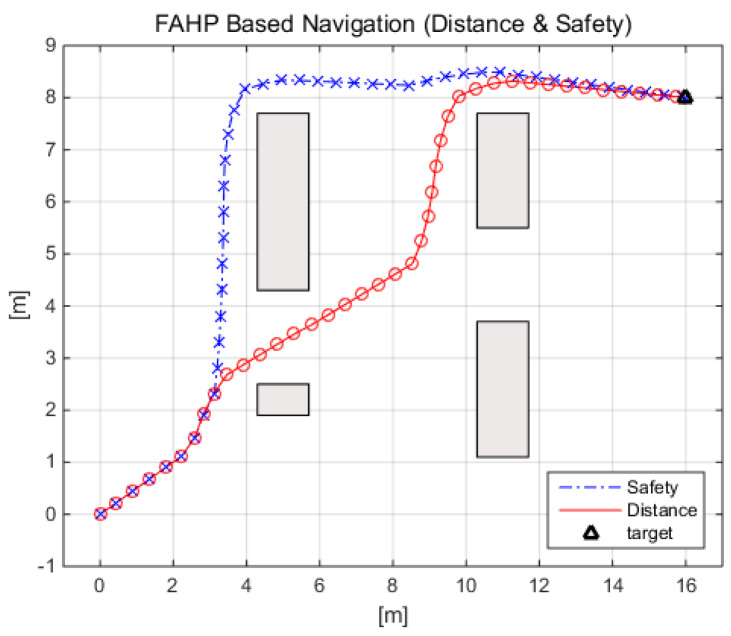
Comparison between short-distance and high-safety RM-based AHP path planning.

Figure 5 shows the FAHP-based navigation results under two different preferences: ${\mathrm{RM}}_{\mathrm{dist}}$ and ${\mathrm{RM}}_{\mathrm{safety}}$. A mobile robot navigates from the initial position $p_{o}\left(0,0\right)$ to the target position $p_{t}\left(16,8\right)$. The ${\mathrm{RM}}_{\mathrm{dist}}$-based simulation results show 19.75 m for the total traveling distance and 87.85 for the average safety score. On the other hand, the ${\mathrm{RM}}_{\mathrm{safety}}$-based case achieves 22.09 m for the total traveling distance and 97.83 for the average safety score. The simulation results show that the ${\mathrm{RM}}_{\mathrm{dist}}$-based FAHP performs better in terms of the traveling distance and the ${\mathrm{RM}}_{\mathrm{safety}}$-based FAHP outperforms the other in terms of safety. As shown in [20], one advantage of the FAHP is that it can manipulate the navigation path according to the user’s preference; in other words, the navigation path can be adjusted according to the environment of navigation. This feature allows the FAHP-based mobile robot navigation to be used not only in industrial sites, where driving-efficiency is of greater importance, but also in hospital service areas, where collision safety is of greater importance.

### 5.2. Simulation II (FAHP-CGT-Based Multi-Robot Navigation under No Obstacle Condition)

**Figure 6:**
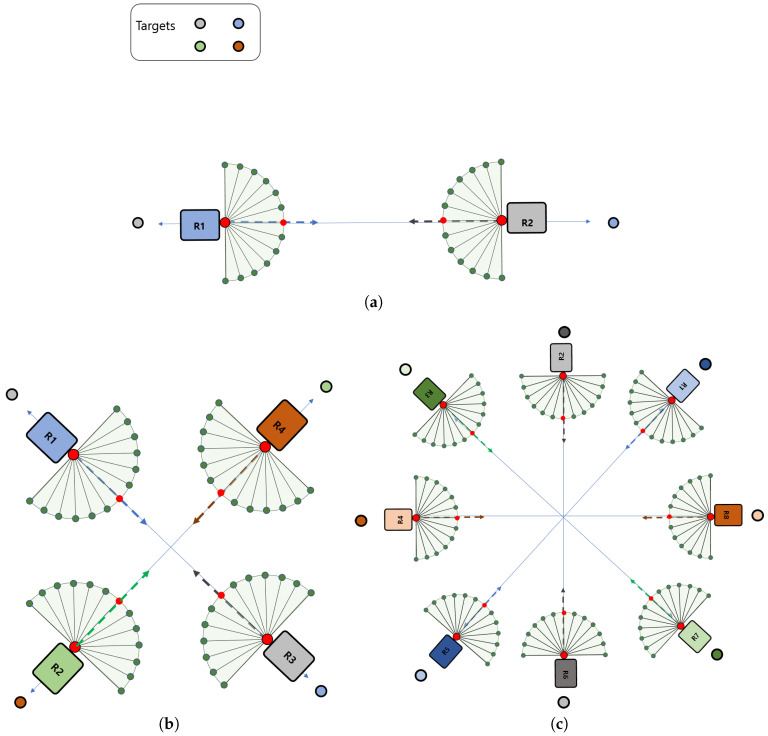
Multi-robot navigation cases: (**a**) two robots case, (**b**) four robots case, (**c**) eight robots case.

In this section, the hybrid navigation algorithm, the FAHP-CGT combined algorithm, is simulated under a different number of robot situations, as shown in Figure 6. Small colored circles represent the target for the corresponding robot; i.e., the target of robot 1 ($R_{1}$) is a blue dot while the gray dot represents $R_{2}$. As Figure 2 displays, the proposed navigation algorithm consists of two parts. Depending on the presence of other robots within the sensing boundary, the algorithm works differently. When there are no other robots within the sensing boundary, only FAHP plans the path, but when the robot meets other robots, the FAHP-CGT algorithm is utilized. In the latter case, all robots communicate with each other to share strategies and corresponding payoffs to make a decision to avoid collision and minimize the loss of payoff.

**Figure 7:**
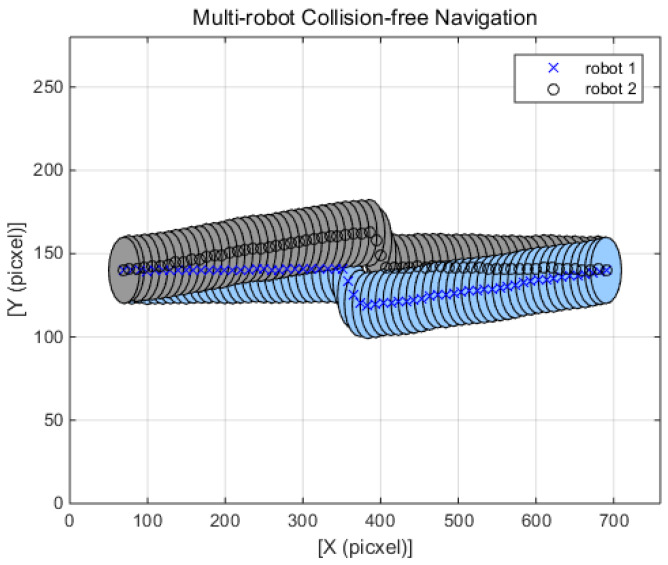
FAHP-CGT algorithm two robot navigation.

**Figure 8:**
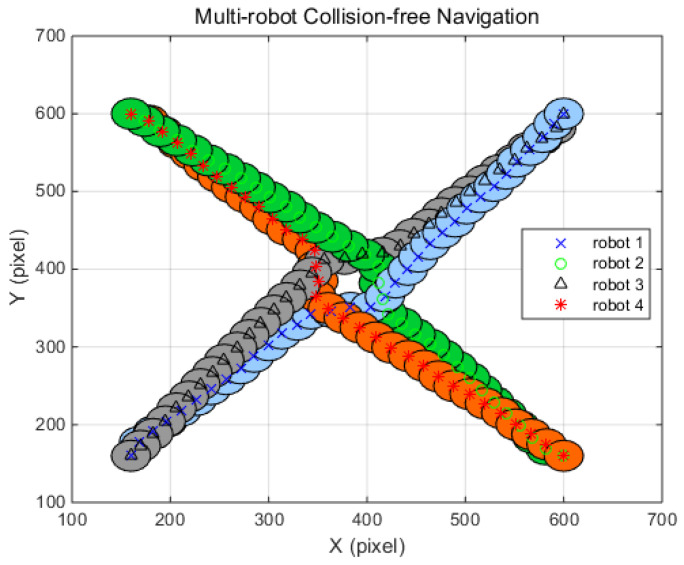
FAHP–cooperative game theory (CGT) algorithm for four-robot navigation.

**Figure 9:**
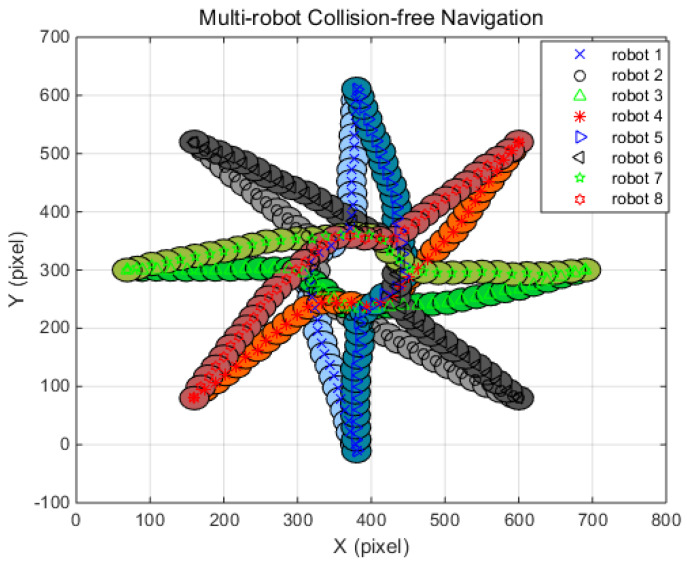
FAHP-CGT algorithm for eight-robot navigation.

**Figure 10:**
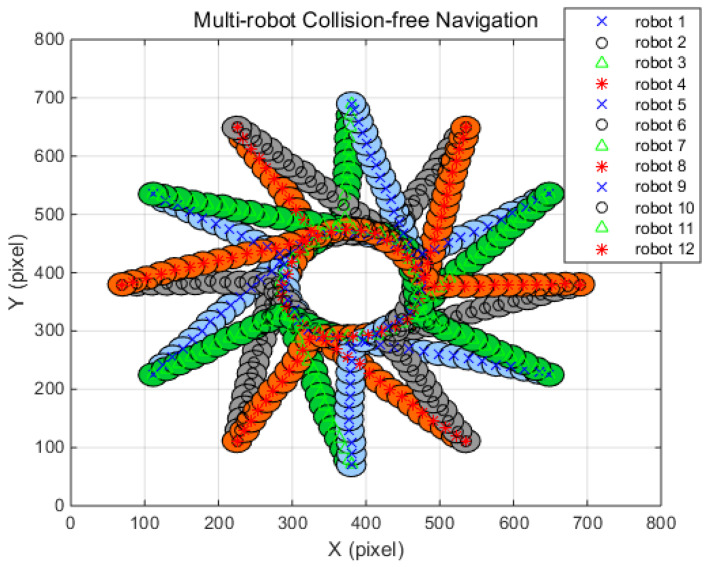
FAHP-CGT algorithm for 12 robot navigation.

In order to demonstrate the performance of the suggested algorithm, the simulation results are compared with the existing multi-robot collision avoidance algorithms, the reciprocal orientation algorithm (ROA) and the shortest distance algorithm (SDA) [42]. For quantitative comparison with ROA and SDA, simulation conditions such as a robot radius of 20 pixels were used, and each robot’s moving distance was set to be the same as those of the reference. In the simulations, the traveling distance and the occurrence of a collision between robots are observed where no obstacles are met during the traveling. In all simulation situations, initial positions and target positions are defined so that the robots can drive in opposite directions. Therefore, a collision between robots is inevitable. Table 4, Table 5 and Table 6 show the navigation conditions, such as initial positions and target positions, of the robots, and the simulation results of two, four, eight, and 12 robots, respectively. In addition, the path corresponding to each simulation case is shown in Figure 7, Figure 8, Figure 9 and Figure 10, respectively. The simulation results show that the suggested algorithm successfully guides the robot to the target without any collision. When the results of the suggested algorithm are compared with ROA and SDA, the results for two and four robots show that the presented algorithm is superior to ROA and SDA. However, the simulation results for eight and 12 robots outperformed ROA but were inferior to SDA. Notably, the performance differences between FAHP-CGT and SDA for eight and 12 robots are only 0.5% and 0.45%. From the simulation results, the proposed algorithm showed a superior or similar performance to the conventional navigation algorithms.

Figure 7 displays the two robot-based navigation performance. In this scenario, two robots switch places with each other; i.e., a robot (named robot 1) moves to the position of another robot (named robot 2), and robot 2 goes to robot 1’s original position. Since there are no obstacles on the path to the target position, the two robots should drive in a straight line until they meet each other. In the middle position of the travel, the two robots interact and decide on where to move for collision-free navigation. Figure 7 shows that the hybrid algorithm-based solution successfully navigates while minimizing the loss of the payoffs for both robots. The traveling distances of robot 1 and robot 2 are 630.09 pixels, 630.42 pixels, respectively. Moreover, the traveling distance results show that only 1.7% is added to the original travel distance while avoiding the collision. When compared with the ROA and SDA algorithm, the performance of the FAHP-CTG is better in terms of travel distance efficiency.

Figure 8, Figure 9 and Figure 10 display the simulation results under the four, eight, and 12 robot navigation conditions. In all simulation scenarios, all the robots navigate to the target following a straight line until they encounter other robots with FAHP. Once multiple robots meet each other, the strategies and related payoffs are shared to make a safe and efficient decision using the FAHP-CGT algorithm. The results show that multiple robots successfully change their direction to avoid collision and move to the targets in all situations. In Table 4, Table 5 and Table 6 the distances of travel for each robot are given. As the robot’s number is increased, the traveling distance is increased. In the four-robot simulations, the proposed algorithm’s result in terms of the average distance increase was 3.3%, which is superior to the values of 6.6% and 4.7% for comparison. On the other hand, in the eight-robot simulation scenario, the proposed method’s results was 5.7%, showing better performance than ROA and worse performance than SDA. The simulation results confirm that the proposed algorithm has superior performance to the other methods (ROA, SDA) in terms of collision-avoidance and driving distance efficiency for at least four robots. However, as the number of the robot increases, the performance of the suggested algorithm has similar performance with SDA. However, in a real-world situation, the number of the interacting robots is limited; therefore, the successful performance of the FAHP-CGT based algorithm is proven.

### 5.3. Simulation III (FAHP-CGT Based Multi-Robot Navigation under a Warehouse Environment)

**Figure 11:**
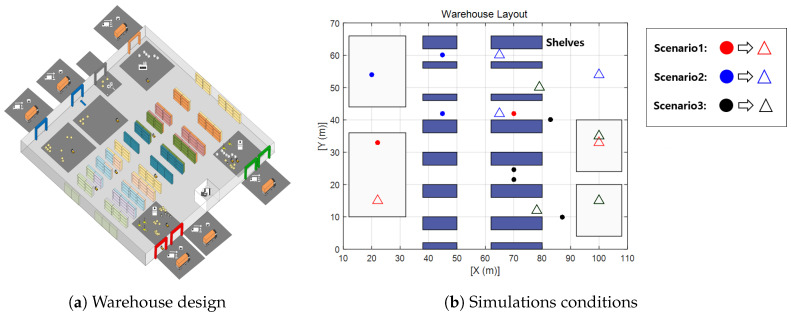
Warehouse design and simulation layout design.

In the previous section, multi-robot navigation under the no-obstacles condition was investigated. However, the actual multi-robot operating environment is more complex. Multi-robot systems can be deployed in several sites such as smart factories, autonomous parking systems, warehouses, and even highly automated hospitals. In this simulation, the performance of the suggested algorithm is tested on a warehouse layout design. In the simulations, three scenarios have been defined that are likely to occur in an environment in which multiple robots work together. Figure 11 shows the warehouse design [43] and the simplified version of the warehouse design—a simulation layout. The first scenario is designed so that two robots intersect each other $(R_{1}$ and $R_{2})$, the second simulation covers three robots crossing $(R_{3}$, $R_{4}$, and $R_{5})$, and the final simulation deals with crossing scenarios of four robots $(R_{6}$, $R_{7}$, $R_{8}$, and $R_{9})$. The purpose of the suggested algorithm is to make a safe and efficient path-generation method. Therefore, in all scenarios, collisions between robots are designed to be inevitable. For the reproduction and comparison, the simulation conditions are given in the table. Furthermore, the radius of the robot is assumed to be 1.1 m. From Figure 11b, colored circles mean the starting position of each robot and the empty triangles represent target positions. Red, blue, and black colors represent scenarios 1, 2, and 3, respectively, and the corresponding positions are provided in Table 7.

**Figure 12:**
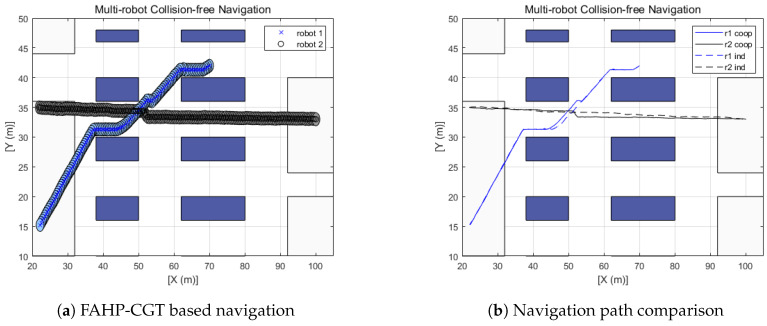
Scenario 1: Two-robot navigation.

**Figure 13:**
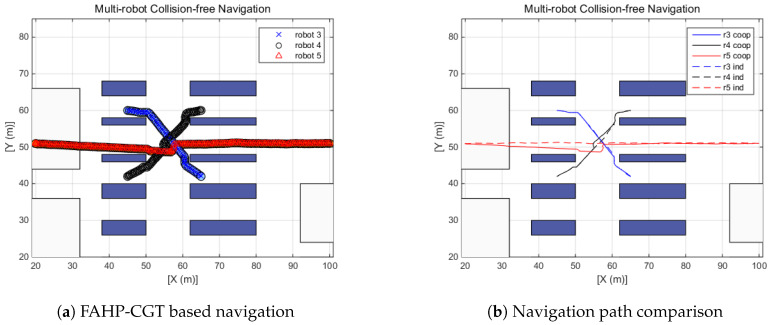
Scenario 2: Three-robot navigation.

**Figure 14:**
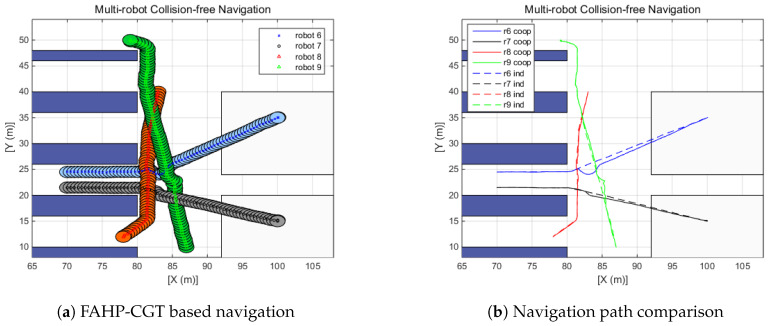
Scenario 3: Four-robot navigation.

The left side plots of Figure 12, Figure 13 and Figure 14 display the trajectory of multi-robot collision-free navigation using the proposed algorithm. The right side plots of Figure 12, Figure 13 and Figure 14 show the comparison between the original travel path (when only one robot navigates to the target by means of FAHP) and the suggested algorithm’s path (when robots meet together on the way to the target). All the simulation results show that there was no collision between robots. Furthermore, the travel distance of the multi-robot operating situation is increased, as shown in Table 8. Increased travel distances are attributed to the regeneration of paths to avoid collisions between robots. Furthermore, the amount is relatively small, at below 5.25%. In the multi-robot navigation problem, the smaller the distance increase rate due to path regeneration, the higher the achieved working efficiency. In these scenarios, the distance increases of three defined scenarios are 1.15%, 1.63%, and 2.0%, respectively.

## 6. Conclusions

In this paper, a multiple mobile robot navigation strategy based on a multi-objective decision-making framework and Fuzzy Analytic Hierarchy Process has been studied. The main advantage of FAHP is that decisions are made through the relative importance of the considerations. Therefore, the robot can be easily operated according to the user’s preference for the objectives under consideration; additionally, the user’s preferences for operating the robot can be applied easily. In this work, FAHP and cooperative game theory were combined to determine the optimal decision situation for multiple robots. The main contribution of this study was the proposal of a collision-free optimal path planning method for multiple mobile robots by considering various targets simultaneously, such as the distance to the target, collision safety, and rotation to the target. FAHP-CGT allows each robot to drive without collisions in an environment in which many robots are operated, taking various factors into account. Through simulations, the performance of the proposed navigation algorithm was verified under several scenarios, such as different RMs and numbers of robots and in a warehouse environment. This study was based on numerical simulations to demonstrate the performance of the proposed path planning method. Future research will focus on the path planning method with a real mobile robot in various working environments. 

## Figures and Tables

**Table 1:** Fuzzified Satty’s scale for triangular fuzzy numbers [36].

Intensity of Importance	Definition
(1, 1, 1 + d)	Equal importance of objectives
(3 − d, 3, 3 + d)	Moderate importance of one objective relative to another
(5 − d, 5, 5 + d)	Strong importance of one objective relative to another
(7 − d, 7, 7 + d)	Very strong importance of one objective relative to another
(9 − d, 9, 9)	Extreme importance of one objective relative to another
(x − d, x, x + d), x = 2, 4, 6, 8	Intermediate values between two adjacent judgements

**Table 2:** Random consistency (RC) index [37].

Number of Objectives	*RC*
3	0.58
4	0.90
5	1.12
6	1.24
7	1.32
8	1.41
9	1.45

**Table 3:** Game theory terminology in multi-robot navigation.

Notation	Definition	Multi-Robot Application
n	Number of players	Number of robots
$S_{i}=\left\{s_{i1},\;s_{i2},\cdots ,s_{im}\right\}$	Strategy space of player i	All the candidates for robot i
$s_{i}$	Strategy of player i	Selection of robot i among candidates
$s=(s_{1},\cdots ,s_{n})$	Strategy profile of n players	Each robot’s solution
$s_{-i}=\left(s_{1},\cdots ,s_{i-1},\;s_{i+1},\cdots ,s_{n}\right)$	Strategy profile of n − 1 players	A set without the selection of robot i
**U**	Domain of all possible outcomes	A set of possible benefit for robots
$u=(u_{1},\;u_{2},\cdots ,u_{n})$	Payoffs given to players under strategy s	Benefit of robots
$u_{i}\left(s_{i},s_{-i}\right)$	Payoff to player i under strategy s	Benefit of robot i

**Table 4:** Multi-robot navigation performance comparison (two and four robot cases). ROA: reciprocal orientation algorithm; SDA: shortest distance algorithm.

	Two Robots					Four Robots						
	R${}_{1}$	R${}_{2}$	Average	ROA	SDA	R${}_{1}$	R${}_{2}$	R${}_{3}$	R${}_{4}$	Average	ROA	SDA
Initial position	(70, 140)	(690, 140)				(160, 160)	(600, 160)	(600, 600)	(160, 600)			
Target position	(690, 140)	(70, 140)				(600, 600)	(160, 600)	(160, 160)	(600, 160)			
Travel distance (pixel)	630.09	630.42	630.26	644.00	641.00	641.42	639.23	639.07	642.82	640.63	661.00	649.00
Increased distance(%)	1.6	1.7	1.7	3.9	3.4	3.5	3.1	3.1	3.7	3.3	6.6	4.7

**Table 5:** Multi-robot navigation performance comparison (two and four robot cases).

	Eight Robots										
	R${}_{1}$	R${}_{2}$	R${}_{3}$	R${}_{4}$	R${}_{5}$	R${}_{6}$	R${}_{7}$	R${}_{8}$	Average	ROA	SDA
Initial position	(380, 611)	(160, 520)	(69, 300)	(160, 80)	(380, −11)	(600, 80)	(691, 300)	(600, 520)			
Target position	(380, −11)	(600, 80)	(691, 300)	(600, 520)	(380, 611)	(160, 520)	(69, 300)	(160, 80)			
Travel distance (pixel)	651.26	652.90	657.42	656.89	654.59	658.05	657.24	655.07	655.43	687.00	652.00
Increased distance (%)	5.0	5.3	6.0	5.9	5.6	6.1	6.0	5.7	5.7	10.8	5.2

**Table 6:** Multi-robot navigation performance comparison (two and four robot cases).

	12 Robots														
	R${}_{1}$	R${}_{2}$	R${}_{3}$	R${}_{4}$	R${}_{5}$	R${}_{6}$	R${}_{7}$	R${}_{8}$	R${}_{9}$	R${}_{10}$	R${}_{11}$	R${}_{12}$	Average	ROA	SDA
Travel distance (pixel)	674.15	679.24	679.44	681.68	678.37	676.24	675.31	680.48	679.76	678.30	679.99	675.87	678.04	713.00	675.00
Increased distance (%)	8.73	9.55	9.59	9.95	9.42	9.07	8.92	9.75	9.64	9.40	9.68	9.01	9.36	15.00	8.87

**Table 7:** Simulation conditions.

	R${}_{1}$	R${}_{2}$	R${}_{3}$	R${}_{4}$	R${}_{5}$	R${}_{6}$	R${}_{7}$	R${}_{8}$	R${}_{9}$
Initial position	(70, 42)	(22, 35)	(45, 60)	(45, 42)	(100, 51)	(70, 24.5)	(70, 21.5)	(83, 40)	(78, 12)
Target position	(22, 15)	(100,33)	(65, 42)	(65, 60)	(20, 51)	(100, 35)	(100, 15)	(87, 10)	(79, 50)

**Table 8:** Simulation results of multi-robot navigation.

	Scenario 1		Scenario 2			Scenario 3			
	R${}_{1}$	R${}_{2}$	R${}_{3}$	R${}_{4}$	R${}_{5}$	R${}_{6}$	R${}_{7}$	R${}_{8}$	R${}_{9}$
Original travel distance (m)	58.03	78.00	28.79	28.66	80.04	32.62	31.05	29.53	42.06
Cooperative travel distance (m)	58.95	78.54	28.94	29.18	82.16	34.28	31.35	29.63	42.71
Increased distance (%)	1.6	0.7	0.5	1.8	2.6	5.1	1.0	0.3	1.6
